# iMSC-derived extracellular vesicles and their miRNA cargo influence inflammation and oxidative damage in an in vitro osteoarthritis model

**DOI:** 10.1038/s41419-026-09033-0

**Published:** 2026-06-24

**Authors:** Matilde Balbi, Matteo Rovere, Vanessa Cossu, Daniele Reverberi, Emanuele Quarto, Matteo Formica, Silvia Pontara, Georgina M. Shaw, Paola Ostano, Giovanna Chiorino, Silvia Ravera, Simona Coco, Mary Murphy, Chiara Gentili

**Affiliations:** 1 Department of Experimental Medicine (DIMES), University of Genoa, Genoa, Italy; 2 UOC Research-Scientific Direction, Plesso: Ospedale Policlinico San Martino, IRCCS Azienda Ospedaliera Metropolitana (IRCCS AOM), Genoa, Italy; 3 UOC Clinica Ortopedica, Plesso: Ospedale Policlinico San Martino, IRCCS Azienda Ospedaliera Metropolitana (IRCCS AOM), Genoa, Italy; 4 Regenerative Medicine Institute (REMEDI), University of Galway (UoG), Galway, Ireland; 5 Cancer Genomics Lab, Fondazione Edo ed Elvo Tempia, Biella, Italy Azienda Ospedaliera Metropolitana (IRCCS AOM), Genoa, Italy; 6 Plesso: Ospedale Policlinico San Martino, IRCCS Azienda Ospedaliera Metropolitana (IRCCS AOM), Genoa, Italy; 7 UOS Tumori Polmonari, Plesso: Ospedale Policlinico San Martino, Oncologia Medica IRCCS Azienda Ospedaliera Metropolitana (IRCCS AOM), Genoa, Italy; 8 UOC Oncologia Cellulare, Plesso: Ospedale Policlinico San Martino, IRCCS Azienda Ospedaliera Metropolitana (IRCCS AOM), Genoa, Italy

**Keywords:** Multipotent stem cells, Regeneration, Induced pluripotent stem cells

## Abstract

Osteoarthritis is a multifactorial chronic joint disease characterized by progressive cartilage degradation and inflammation. Since there is no effective cure, emerging therapeutic approaches, such as mesenchymal stromal cells (MSCs) transplantation, are currently under investigation. However, the clinical translation of MSC-based therapies is hampered by several limitations, such as donor-dependent variability and heterogeneity related to tissue sources. To address these issues, MSCs derived from induced pluripotent stem cells (iMSCs) have been proposed as a more standardized and scalable alternative. Due to the risks of cell-based therapy, extracellular vesicles (EVs), particularly iMSC-EVs (iEVs), could represent a promising cell-free approach for OA treatment. The present study aimed at characterizing iMSC-derived EVs and evaluating their functional role in modulating inflammatory responses and redox balance in an in vitro OA model. Notably, recent evidence highlights the central role of EV-encapsulated microRNAs (EV-miRNAs) in mediating these effects. EVs isolated from iMSC conditioned media were characterized, and their miRNA content was analyzed at different culture passages. Selected miRNAs were subsequently assessed for their biological activity in an in vitro OA model, with a focus on their impact on inflammatory mediators and oxidative stress parameters. Specifically, six miRNAs such as hsa-miR-17-5p, hsa-miR-20a-5p, hsa-miR-21-5p, hsa-miR-29a-3p, hsa-miR-29b-3p, and hsa-miR-29c-3p differentially reflect the anti-inflammatory and antioxidant effects of iMSCs-EVs treatment, suggesting possible synergistic effects. Their combined effect in the in vitro model confirmed their potential modulation in the expression of pro-inflammatory cytokines. Furthermore, their treatment markedly reduced ROS accumulation and oxidative damage, while restoring antioxidant defense systems. These findings support the therapeutic potential of iMSC-derived EVs as a cell-free strategy for OA treatment. The miRNA cargo encapsulated within iEVs appears to play a pivotal role in modulating inflammation and oxidative stress, emphasizing their promise as a novel, minimally invasive approach for disease modification in OA.

**Figure:**
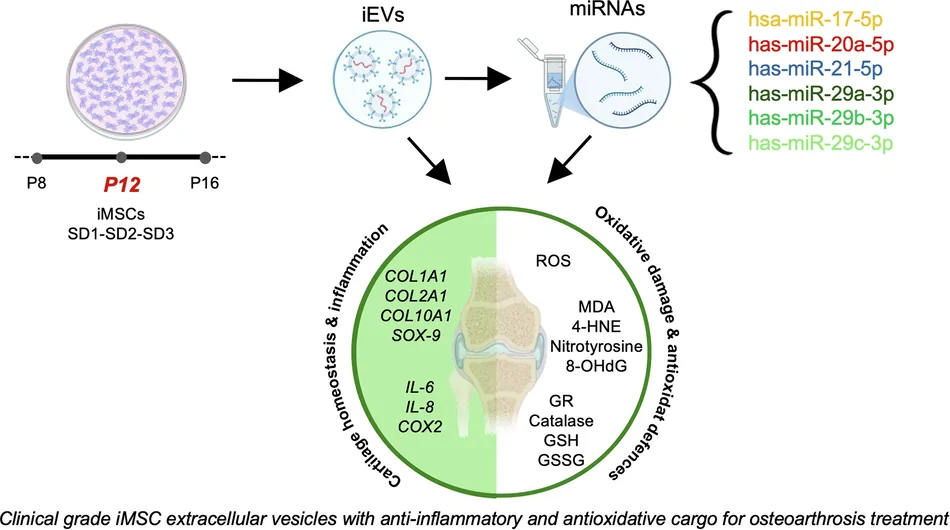

## Introduction

Osteoarthritis (OA) is a multifactorial, degenerative joint disease affecting millions worldwide, with prevalence increasing in aging population [1]. OA is characterized by cartilage breakdown, ligament calcification, and subchondral bone outgrowth, leading to progressive loss of joint functionality [2, 3]. Although its pathogenesis is still unclear, increased production of pro-inflammatory cytokines and redox imbalance are the main triggers of disease progression [3–5]. Current treatments primarily rely on oral or topical nonsteroidal anti-inflammatory drugs (NSAIDs) for symptomatic relief, but they do not alter disease progression [6]. Many approaches have been tested, including drug repurposing (e.g., doxycycline, metformin), intra-articular injections (Platelet Rich Plasma, corticosteroids), and regenerative surgical techniques. However, to date, no treatment capable of significantly reversing the course has been developed [7, 8]. The development of alternative therapeutic approaches still represents an unmet need.

In this context, mesenchymal stromal cells (MSCs) or the direct use of their derived extracellular vesicle (MSC-EVs) have emerged as promising candidates due to their immunomodulatory and anti-inflammatory properties [9].

MSCs are cells of the mesodermal lineage that possess different stemness properties. MSCs can differentiate into mesodermal lineages (e.g., osteocyte, chondrocyte, and adipocyte), making them attractive candidates for restoring joint function [10]. Several clinical trials have demonstrated that MSC-injection ameliorates clinical symptoms, showing chondroprotective effects and positively affects cartilage repair. However, significant limitations associated with cell-based therapies remain [11–14]. These include donor variability, low cell yield, limited scalability, and ethical concerns, all of which hinder clinical translation [15–17].

In contrast, MSC-derived EVs retain comparable therapeutic efficacy while avoiding risks associated with live-cell administration, including immune rejection, chromosomal instability, and tumorigenicity [18]. Since extensive in vitro expansion needed to obtain therapeutic amounts of EVs induces senescence and reduces classical MSC multipotency markers, we propose induced pluripotent stem cell–derived MSCs (iMSCs) as an alternative source. iPSCs exhibit high proliferative and differentiation capacity, and iMSC differentiation yields cells comparable to primary MSCs in morphology, surface phenotype, and lineage potential, while allowing long-term expansion without senescence [19–24].

To address the limitations of MSC-based therapies and support their paracrine mechanism, increasing attention has focused on the MSC secretome and EVs, which retain anti-inflammatory and cartilage supportive activities [25–28].

EVs, a heterogeneous population of membrane-bound particles, are now recognized as promising therapeutic agents and key mediators of intracellular communications. EVs carry biological materials and deliver bioactive cargo, including proteins, lipids, and nucleic acids to recipient cells, with microRNAs (miRNAs) playing a central role by regulating gene stability and translation in target cells [29].

Differences in EV-miRNA cargo can modulate the paracrine effects on target cells and reflect the physiological or pathological state of the originating cells. Specific miRNAs, such 92a-3p and miR-140-5p, promote chondrogenic, inhibit cartilage degradation, delay OA progression in a rat in vivo model, highlighting the potential therapeutic role of these miRNAs in OA [27]. However, the role of iMSC-derived EVs (iEVs) and their cargo in modulating oxidative stress in OA remains largely unexplored.

Based on these premises, this study employed long-term iMSC cultures to identify the optimal window for iEV isolation and to profile their miRNA cargo. In addition, the capacity of iEVs to modulate the secretion of pro-inflammatory cytokines and to activate antioxidant defense mechanisms, reducing reactive oxygen species (ROS)-caused damage, was evaluated. Finally, the functional relevance of the most abundant EV- miRNAs was validated in an in vitro osteoarthritic model.

## Results

### iMSC expansion and iEVs sample collection

**Fig. 1:**
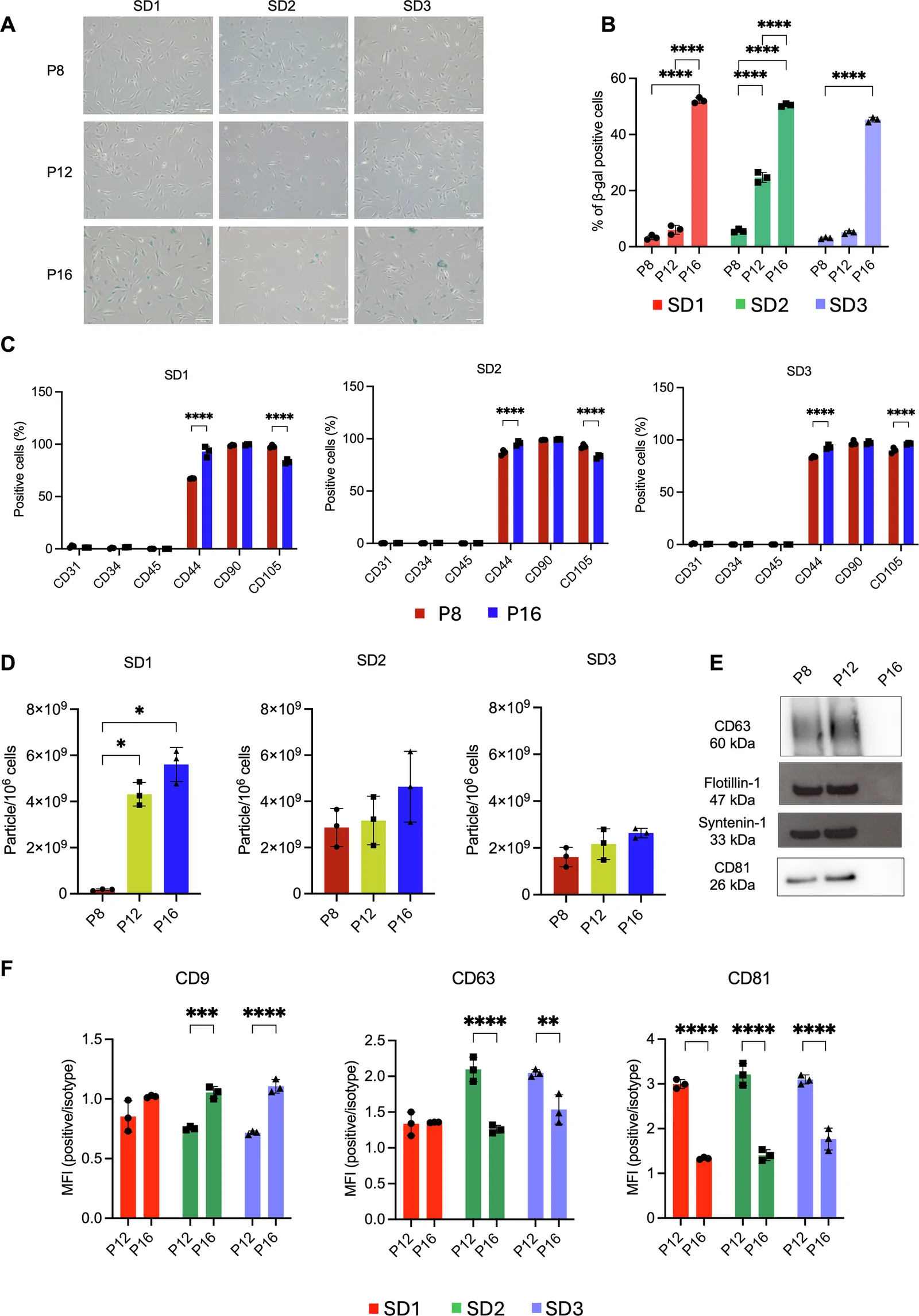
Long-term iMSCs SD1, SD2, SD3 iMSC and iEVs characterization. **A** Representative image of β- galactosidase staining for SD1, SD2, and SD3 at P8, P12, and P16, Scale bar = 250 μm. **B** Quantification of β-galactosidase-positive cells. Data are represented as mean ± SD (*n* = 3). Two-way ANOVA, **** *p*-value < 0.0001. **C** Flow cytometry analysis of iMSCs at culture passage (P) 8 and P16. Histograms show the average percentage of positive events for each marker. Data are represented as mean ± SD (*n* = 3), One-way ANOVA, * *p*-value < 0,05, *** *p*-value < 0.001, **** *p*-value < 0.0001. **D** NTA of particle concentration, expressed as particles/10^6^ cells. Data are represented as mean ± (*n* = 3), One-way ANOVA, *** *p*-value < 0.001, **** *p*-value < 0.0001. **E** Western blot analysis on iEVs isolated at P8, P12, and P16. Specific expressions of CD63, CD81, flotillin-1, and syntenin-1 were investigated. Data are representative of one single cell line. **F** Quantification of flow cytometry analysis of CD9-, CD63-, and CD81- positive events in the CFDA-SE gate. Data are shown as a ratio between mean fluorescence intensity (MFI) of EVs stained with the specific antibody and the corresponding isotype control (relative MFI) (*n* = 3). One-way ANOVA, ** *p*-value < 0.01, *** *p*-value < 0.001, **** *p*-value < 0.0001.

Three iMSCs lines derived from iPSC (SD1, SD2, and SD3) were cultured for about 70 days, from passage P8 to P16. Cumulative doublings were calculated at each passage, and the conditioned medium was collected for iEVs isolation. Cell morphology and senescence were assessed at P8, P12, and P16, corresponding to early, middle, and late stages, respectively. Analysis of the growth curves revealed heterogeneity among the three cell lines: SD1 proliferated faster and maintained exponential growth longer than SD2 and SD3 (55 vs. 45 days for SD2 and SD3) (Fig. S1 and Table 1). Cellular senescence, evaluated by β-galactosidase staining, revealed a marked increase in β-gal+ cells at P16 in SD1 and SD3, whereas SD2 showed an early onset of senescence at P12 (SD1 P8: 3.40 ± 0.79; P12: 5.87 ± 1.68; P16 51.56 ± 0.34; SD2 P8: 5.87 ± 0.46; P12: 24.61 ± 1.80; P16: 50.51 ± 0.51; SD3 P8: 3.11 ± 0.29; P12: 5.12 ± 0.50; P16: 45.81 ± 0.36) (Fig. 1A, B). Phenotypic stability was evaluated at P8 and P16. Mesenchymal markers CD44, CD90, and CD105 were retained, while hematopoietic markers (CD31, CD34, CD45) remained absent (Fig. 1C). CD44 expression increased over time, CD90 remained high, and CD105 decreased in SD1 and SD2 but increased in SD3. No differences were observed in the expression of hematopoietic markers, whose expression remained absent during all culture times.

**Table 1:** iMSC cumulative doubling at culture passage (P-) 8, 12, and 16.

	P8	P12	P16
SD1	4.31 ± 3.45	10.77 ± 4.56	13.10 ± 4.57
SD2	3.17 ± 2.56	8.21 ± 4.89	9.21 ± 2.56
SD3	3.53 ± 3.98	6.69 ± 3.67	7.49 ± 5.67

Data are reported as mean ± SD (*n* = 3).

iEVs were isolated and characterized according to MISEV guidelines [30]. Protein concentration and particle numbers increased over time across all lines (Figs. S1B, C1D and Table 2). In addition, iEV phenotype by western blot, highlighted a reduced expression of all the markers considered (i.e., CD63, Flotillin-1, Syntenin-1, and CD81) in the late culture passages (Fig. 1E). Flow cytometry confirmed the decreased expression of both CD63 and CD81 during the culture time, while CD9 expression slightly increased over time (Fig. 1F and Table 3). Overall, prolonged culture leads to a progressive loss of iEVs phenotypic markers, suggesting that increased protein secretion at late passages does not reflect functional EVs abundance.

**Table 2:** NTA of the size of EVs isolated from SD1, SD2, and SD3.

	Mean size	Peak size
SD1	150.40 ± 2.64 nm	129.78 ± 8.78 nm
SD2	149.53 ± 4.95 nm	129.08 ± 4.66 nm
SD3	139.03 ± 8.50 nm	120.00 ± 5.70 nm

Data are reported as mean ± SD (*n* = 4).

**Table 3:** Percentage of CDFA-SE-positive events in the considered three-dimensional gates (EVs <100 nm, 100 nm < EVs <160 nm, and 160 nm < EVs < 900 nm).

	EVs < 100 nm	100 nm < EVs < 160 nm	160 nm < EVs < 900 nm
SD1	15.77 ± 4.71%	62.90 ± 4.28%	20.93 ± 8.61%
SD2	19.97 ± 2.55%	54.60 ± 12.54%	25.37 ± 11.84%
SD3	20.80 ± 1.91%	64.47 ± 2.15%	14.73 ± 2.31%

Data are reported as the mean ± SD (*n* = 3).

### iEVs treatment shows anti-inflammatory effects and maintains chondrogenicity on an in vitro *OA* model

**Fig. 2:**
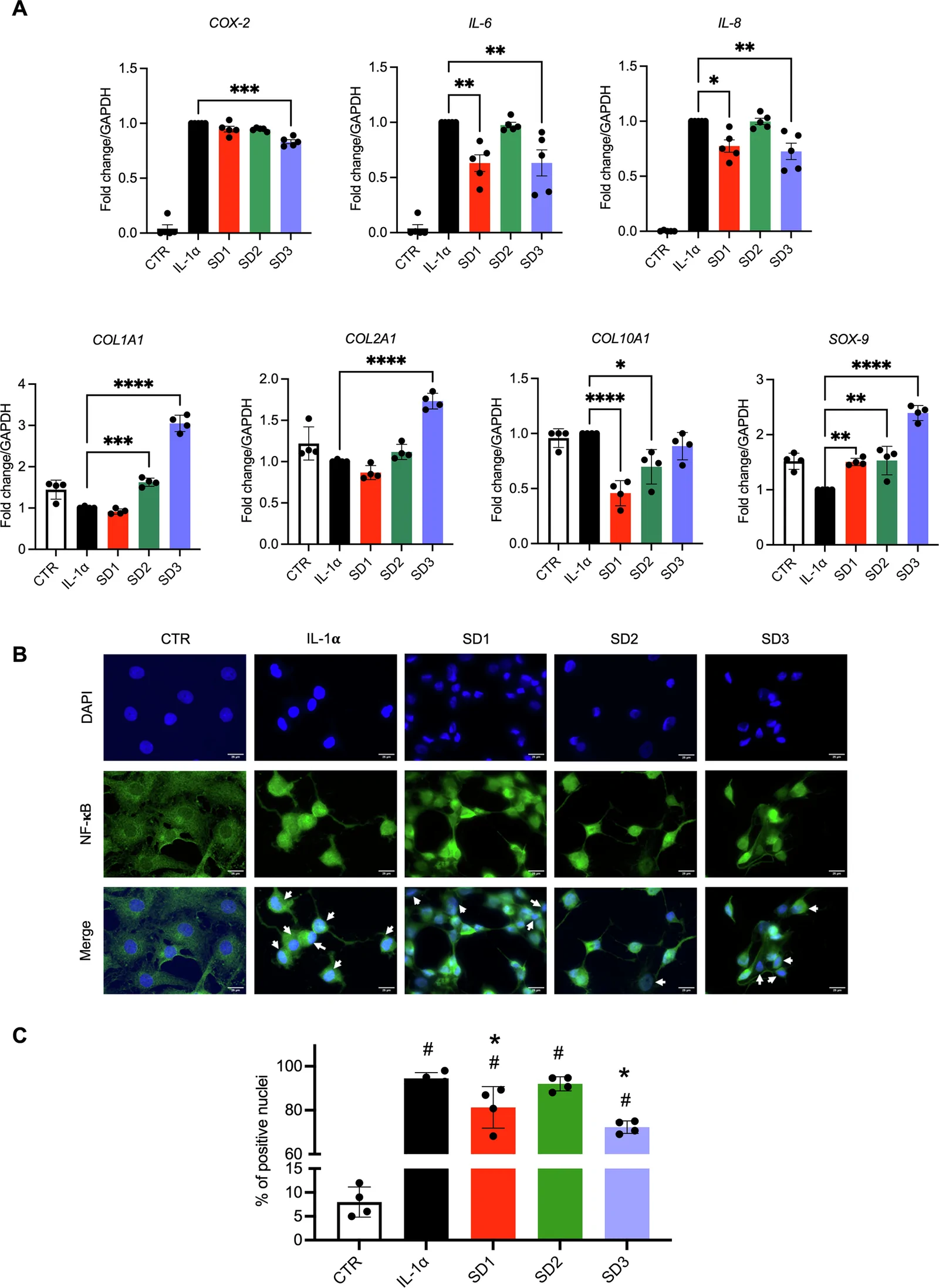
P12 iEVs treatment on human articular chondrocytes biological effect. **A** Quantitative real-time PCR for the expression of IL-6, IL-8, and COX-2, COL1A1, COL1A1, COL10A1 and SOX-9. Data are represented as mean ± SD (*n* = 4, 5). One-way ANOVA, # *p*-value < 0.0005 vs CTR, * *p*-value < 0.05 vs IL-1α. CTR negative control. **B** NFkB nuclear translocation in OA hACs in response to iEVs treatment. White arrows indicate negative cells. Images are representative of four independent experiments. **C** Quantification of NF-kB nuclear translocation, calculated as the number of cells with positive nuclei on the total cell number. Data are represented as mean ± SD (*n* = 4), One-way ANOVA. # *p*-value < 0.0005 vs CTR, * *p*-value < 0.01 vs IL-1α. CTR negative control.

IL-1α-primed hACs (100 U/mL) recapitulated OA-like inflammation, inducing NF-kB nuclear translocation and increased expression of *COX-2, IL-6*, and *IL-8*. Consistent with existing literature, MSC-EVs inhibited NF-kB nuclear translocation, thereby reducing the inflammatory response [31, 32]. Based on yield and phenotypic preservation, middle-passage iEVs (P12) were selected for functional studies. IL-1α–treated hACs were exposed to iEVs from SD1, SD2, and SD3, revealing heterogeneous responses: SD1- and SD3-derived iEVs significantly reduced IL-6 and IL-8, whereas SD2-iEVs showed no effect. COX-2 was reduced only by SD3-iEVs (Fig. 2A).

Chondrogenic markers (i.e., *COL1A1*, *COL2A1*, *COL10A1, SOX-9)* were assesses by qPCR (Fig. 2A). *COL1A1* was significantly upregulated in IL-1α-primed hACs treated with SD3 and SD2- derived EVs, while *COL2A1*, a classical collagen type present in the articular cartilage matrix, increased only after SD3-EVs treatment. *COL10A1*, a collagen typically expressed during chondrocyte hypertrophy and osteogenic differentiation, was not modulated following treatment with SD3-EVs, whereas EVs derived from the other two cell lines led to a significant reduction in its expression. Notably, *SOX-9* expression was upregulated by EVs isolated from all three cell lines. Taken together, these findings indicate that the pro-chondrogenic effects were mainly enhanced by EVs derived from SD1 and SD3, while SD2-EVs exhibited limited pro-chondrogenic effects. This observation correlates with the previously reported lack of anti-inflammatory activity of SD2-derived EVs and underscores the considerable batch-to-batch variability observed among iEVs.

### iEV treatment reduces IL-1α-induced NF-κB nuclear translocation

NF-κB is a key regulator of inflammation, controlling cytokine expression and inflammatory mediator production in osteoarthritis (OA). To evaluate whether iEVs modulate NF-κB activity, its subcellular localization was analyzed by immunofluorescence. IL-1α–induced nuclear translocation of NF-κB was partially reduced by SD1- and SD3-iEVs (−13.67% and −24.01%, respectively), while SD2-iEVs had no significant effect (−2.72%; Fig. 2B, C). This heterogeneity is consistent with the previously described differential anti-inflammatory effects.

### iEVs treatment shows a strong activation of antioxidant defenses in an in vitro OA model

**Fig. 3:**
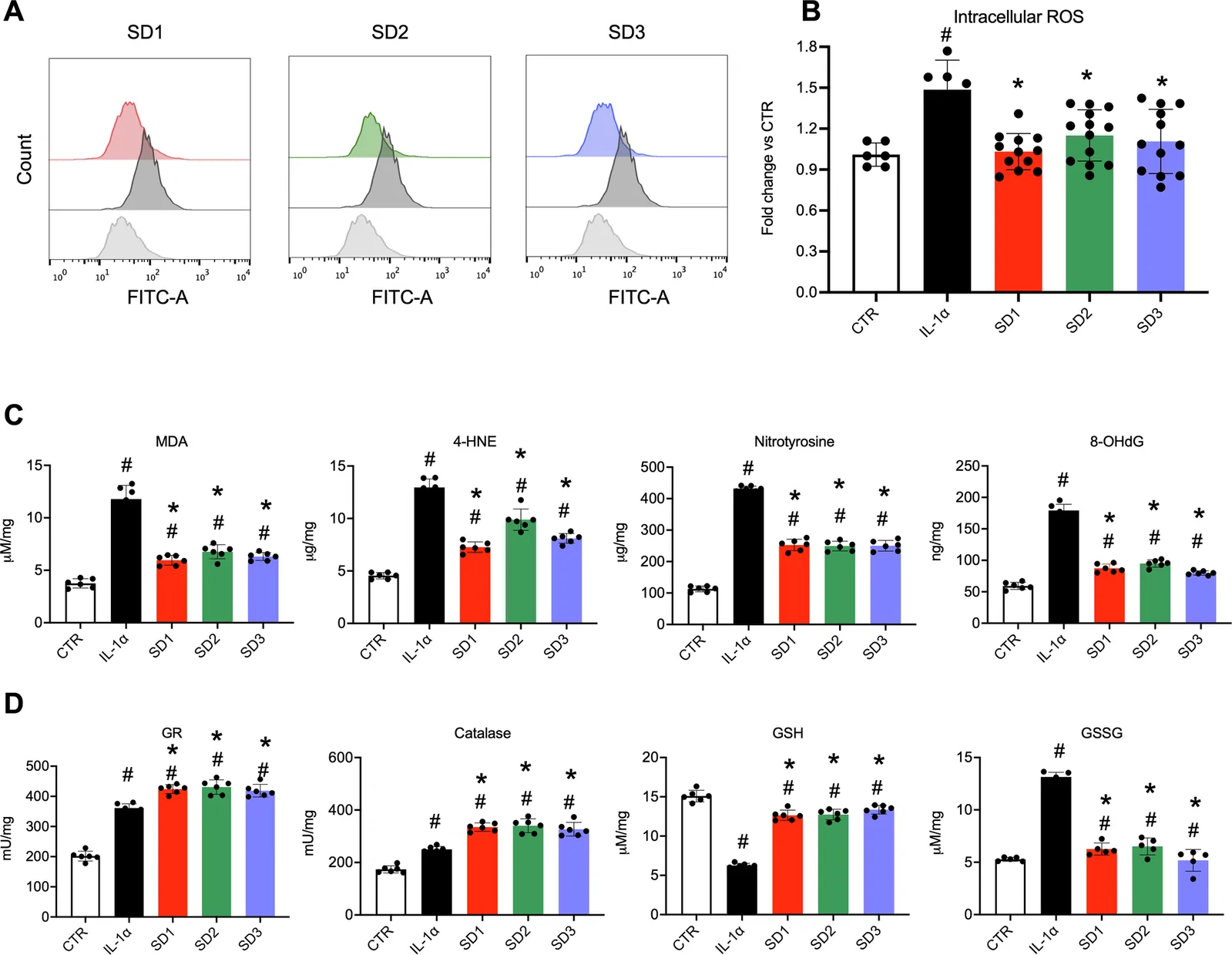
Redox potential of P12 iEVs treatment on human articular chondrocytes. **A** Representative histogram reporting flow cytometry analysis of intracellular reactive oxygen species (ROS) of OA hACs treated with SD1-EVs (red), SD2-EVs (green), and SD3-EVs (blue). The light gray curve represents the negative control, and the dark gray curve positive control. **B** Flow cytometry quantification of intracellular ROS. Data are represented as Fold change of DCF mean fluorescence intensity with respect to CTR (*n* = 11), One-way ANOVA, # *p*-value < 0.0005 vs CTR, * *p*-value < 0.05 vs IL-1α. CTR negative control. **C** Analysis of oxidative damage accumulation on OA hACs treated with iEVs. Data are represented as mean ± SD for the following oxidative damage accumulation markers: MDA, 4HNE, nitro-tyrosine, 8OHdG (*n* = 6). One-way ANOVA, # *p*-value < 0.0005 vs CTR, * *p*-value < 0.005 vs IL-1α. CTR: Negative control. **D** Analysis of antioxidant defense activity on OA hACs treated with iEVs. Data are represented as mean ± SD for the following oxidative damage accumulation markers: GR, Catalase, GSH, and GSSG (n = 6). One-way ANOVA, # *p*-value < 0.0005 vs CTR, * *p*-value < 0.005 vs IL-1α. CTR negative control, 4-HNE 4-hydroxynonenal, 8-OHdG 8-hydroxy-2′-deoxyguanosine, GR glutathione reductase, GSH reduced glutathione, GSSG oxidized glutathione, MDA malondialdehyde, ROS reactive oxygen species.

Several studies have highlighted the pivotal role of ROS in driving cartilage damage and facilitating OA progression [33–35]. Thus, markers of oxidative damage and antioxidant defenses have been evaluated to investigate the impact of iEV treatment on hACs redox homeostasis. Exposure to IL-1α resulted in a two-fold increase in ROS levels compared to the control conditions (Fig. 3A, B). Notably, treatment with iEVs effectively mitigated this oxidative stress, restoring ROS levels to values comparable to the control group (Fig. 3A, B), suggesting a possible reduction in oxidative damage accumulation.

Supporting this, chondrocytes exposed to IL-1α and subsequently treated with iEVs exhibited a significant reduction in oxidative damage markers, including lipid peroxidation, DNA damage, and protein oxidation, as evidenced by decreased levels of MDA and 4-HNE, 8-OHdG, and nitrotyrosine, respectively (Fig. 3C).

These improvements in redox balance and protection from oxidative damage were associated with an upregulation of antioxidant enzymatic activities. Indeed, although IL-1α stimulation alone already increased GR and catalase activity, iEVs treatment further enhanced these activities, reflecting an increase in GSH levels (Fig. 3D). Specifically, treatment with IL-1α alone decreased GSH levels compared to the control, suggesting that the higher consumption was an attempt to counteract the ROS increase. In contrast, the iEVs addition resulted in a smaller decrease in GSH levels with respect to the control, likely due to increased GR activity, which ensures better antioxidant defense.

Collectively, these findings indicate that iEV treatment improves cellular redox status and protects against oxidative stress by enhancing antioxidant defense machinery.

### iMSCs culture passage influences iEV miRNA cargo

**Fig. 4:**
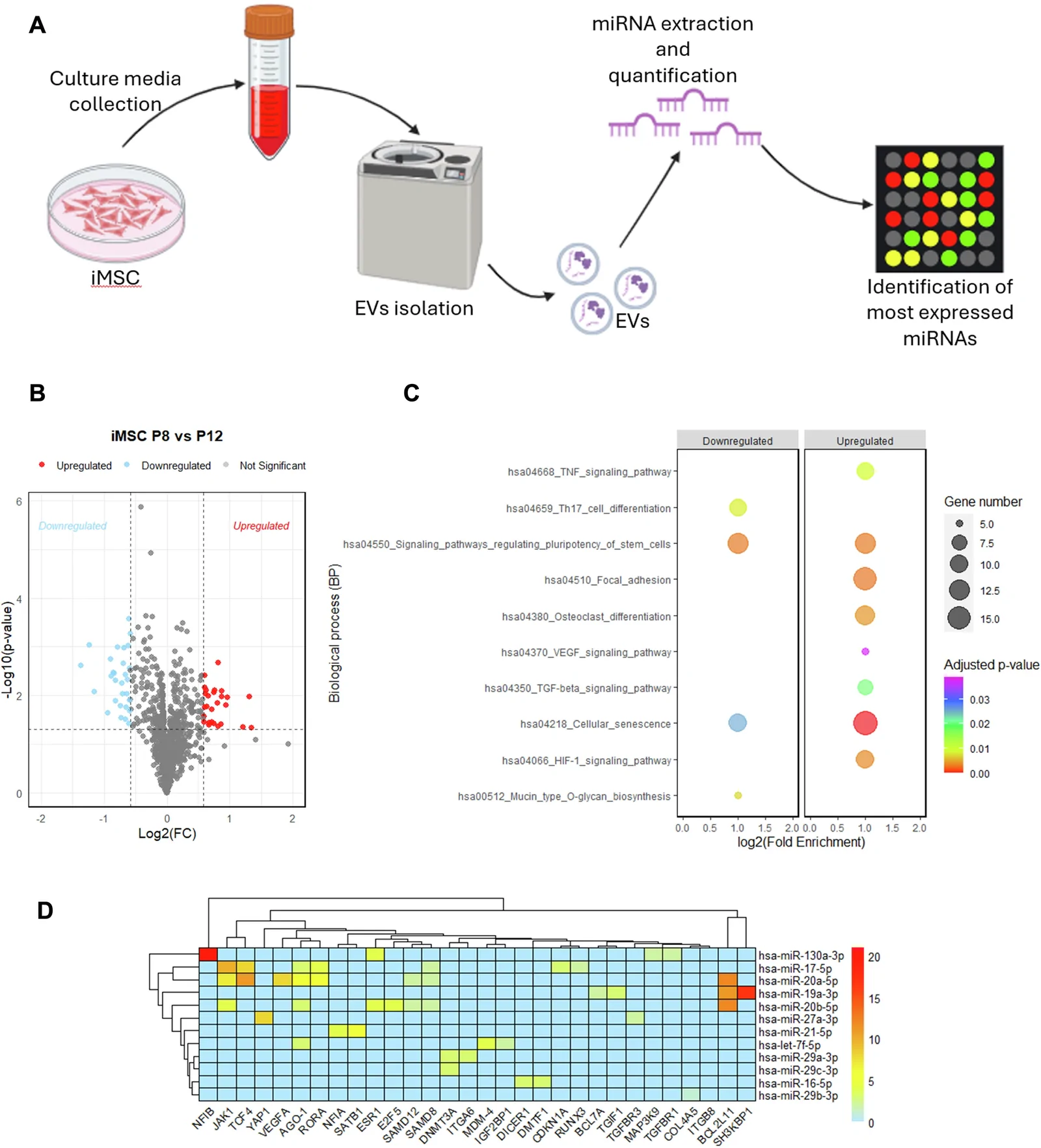
iMSCs culture passage influences iEVs miRNA cargo. **A** Experimental design. **B** Volcano plot showing differently expressed miRNAs in P8-isolated iEVs (blue) compared to P12-isolated iEVs (red). **C** Bubble plot of KEGG pathways regulated by expressed miRNAs analyzed with DIANA miRPath 3.0. **D** Heatmap showing target genes regulated by expressed miRNAs, analyzed with miRsystem. The color scale is representative of the number of hits.

The biological effects of EVs result from the combined activity of various molecular components, including non-coding RNAs such as miRNAs. MiRNAs can influence the outcome of EV interactions with recipient cells by regulating the expression of target gene transcripts. To investigate how miRNA cargo variates along culture time, we analyzed iEV miRNA content at different time points by profiling the expression of 2549 miRNAs (Fig. 4A). Surprisingly, even though BiCinchoninic Acid (BCA) and NTA did not reveal a decreased iEV secretion at P16, their miRNA cargo was not detectable at the late culture stage, in line with the loss of classical iEVs phenotypic markers observed at P16 (Table 4). Based on the previous data, we focused on P8 and P12 from SD1 and SD3 cells, excluding SD2 due to its pronounced cellular senescence observed during culture. In addition, to identify miRNAs associated with anti-inflammatory and antioxidant effects at the middle passage, we performed the analysis in biological duplicates for both cell types and combined the two datasets to compare the EV content between P8 and P12.

**Table 4:** Quantification of iEVs-miRNAs content.

	iMSC culture passage	Concentration (ng/μl)
SD1	P8	295.08
	P12	278.95
	P16	ND
SD2	P8	167.35
	P12	ND
	P16	ND
SD3	P8	121.20
	P12	169.65
	P16	ND

Data are representative of one experiment and are expressed as ng/ μl of RNA extracted from iEVs (ND: not determined).

Notably, we distinguished 29 upregulated and 25 downregulated miRNAs in P12-EVs *versus* P8-EVs (Fig. 4B). Among the upregulated miRNAs, more than half (15/29) are involved in cellular senescence, and focal adhesion remodeling, while others are implicated in the regulation of TGF-β and TNF-α signaling pathways. On the other hand, the downregulated miRNAs were mainly related to the regulation of cellular senescence and stem cell pluripotency (Fig. 4C).

Interestingly most of the targets of the up-regulated miRNAs in P12 iEVs are involved in cartilage degradation, such as *JAK-1* (Janus kinase 1)*, TCF-4* (transcription factor 4), *YAP-1* (Yes1 associated transcription regulator), and *RORα* (RAR-related orphan receptor alpha), suggesting a potential inhibition of genes that can induce cartilage damage [36].

Pro-angiogenic genes such as *VEGF-A* (Vascular endothelial growth factor A), usually involved in the process of cartilage degradation [37], were targeted. Moreover, several miRNA target transcripts were associated with the transcription of proteins related to miRNA biogenesis, such as *DICER* and *AGO-1* (Argonaute RISC component 1), pointing to a possible dysregulation in miRNA production within recipient cells following EV treatment [38]. Intriguingly, transcripts related to apoptosis and senescence-related proteins such as *BCL2L11* (BCL-2 interacting mediator of cell death) and *BCL7A* (BAF Chromatin Remodeling Complex Subunit) are among the targets of the identified miRNAs, indicating a potential effect in reducing senescence (Fig. 4D).

### miRNA iEVs cargo is involved in cartilage homeostasis

**Fig. 5:**
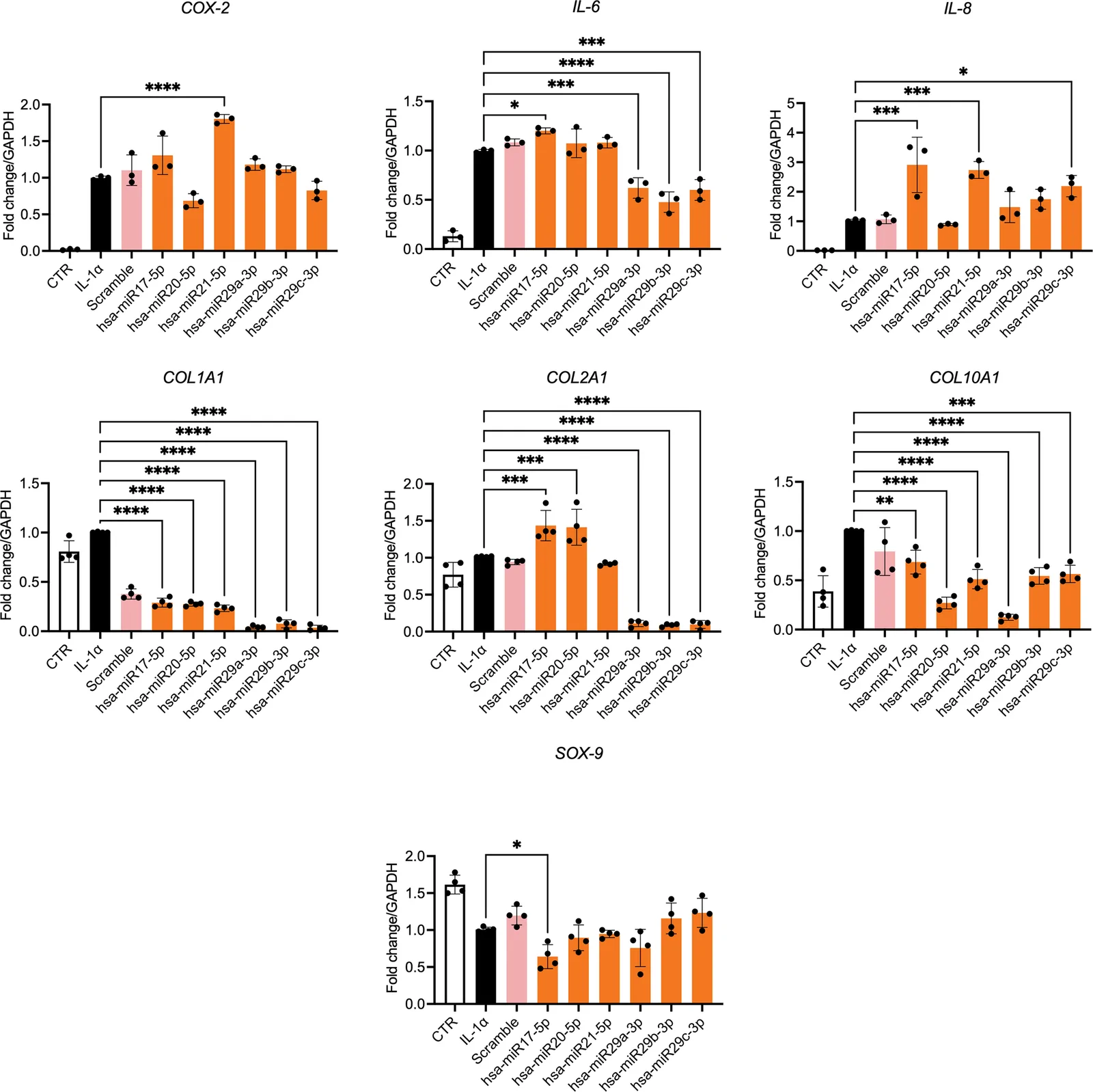
iEVs miRNA cargo is involved in cartilage homeostasis. Human articular chondrocytes were transfected with 100 nM hsa-miR17-5p, hsa-miR20-5p, hsa-miR21-5p, hsa-miR29a-3p, hsa-miR29b-3p, hsa-miR29c-3p or with scramble control. Graphs show the relative expression of target genes *COX2*, *IL-6*, *IL-8*, *COL1A1*, *COL2A1*, *COL10A1*, and *SOX9* analyzed by quantitative real-time PCR. Data are shown as mean ± SD, (*n* = 4). One-way ANOVA, * *p*-value < 0.05, ** *p*-value < 0.01, *** *p*-value < 0.001, **** *p*-value < 0.0001. CTR negative control.

Based on gene ontology analysis, we selected six miRNAs targeting cartilage-related genes (i.e., hsa-miR17-p, hsa-miR20-5p, hsa-miR21-5p, hsa-miR29a-3p, hsa-miR29b-3p, hsa-miR29c-3p). The expression changes induced by these miRNAs were evaluated on the OA in vitro model to assess their potential role in mediating iEV-dependent anti-inflammatory and redox effects. hACs primed with IL-1α were transfected with a single miRNA, and both anti-inflammatory effects and redox potential were evaluated. Specifically, IL-1α-induced *COX-2* expression was significantly enhanced following miR17-5p and hsa-miR21-5p transfection, while it was significantly reduced in response to hsa-miR20-5p. No significant changes in *COX-2* expression were observed after transfection with hsa-miR29a-3p, hsa-miR29b-3p, and hsa-miR29c-3p. In contrast, IL-1α-induced *IL-6* expression was significantly reduced after hsa-miR29a-3p, hsa-miR29b-3p, and hsa-miR29c-3p transfection. Furthermore, hsa-miR17-5p, hsa-miR21-5p, and hsa-miR29c-3p transfection led to a significant increase of *IL-*8 expression (Fig. 5A).

Overall, miRNA exposure displayed heterogenous effects on the expression of cartilage homeostatic markers. *COL1A1* and *COL2A1* gene expressions were downregulated following mir-29 family exposure, while hsa-miR-17-5p and hsa-miR-20-5p led to a significant increase in *COL2A1* level. Interestingly, the pro-osteogenic collagen isoform *COL10A1* was downregulated by all tested miRNAs. No significant changes in *SOX-9* expression were observed, except for a reduction induced by sas-miR-17-5p (Fig. 5B). Overall, these data indicate a heterogeneous effect of miRNAs, suggesting no single miRNA acts specifically to inhibit inflammation in hACs; rather, they may exert a synergic regulatory effect.

### miRNA treatment effects on ROS accumulation and antioxidant effect in an in vitro *OA* model

**Fig. 6:**
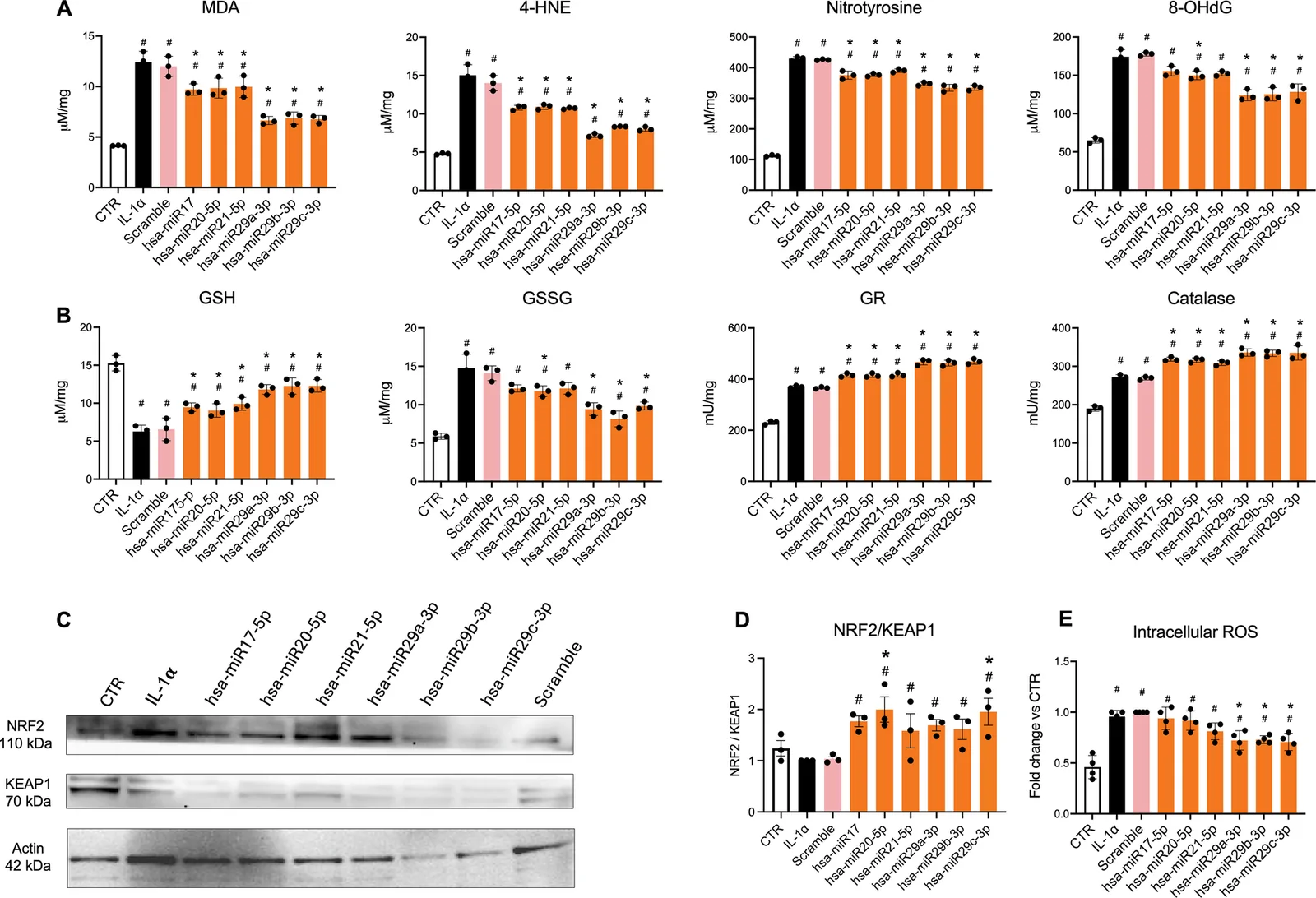
iEV miRNA cargo is involved in the regulation of redox balance in chondrocytes. **A** Analysis of oxidative damage accumulation on OA hACs transfected with single hsa-miR17-5p, hsa-miR20 - 5p, hsa-miR21-5p, hsa-miR29a-3p, hsa-miR29b-3p, hsa-miR29c-3p, or with scramble control (100 nM). Data are represented as mean ± SD for the following oxidative damage accumulation markers: MDA, 4HNE, nitrotyrosine, 8OHdG (*n* = 3). One-way ANOVA, # *p*-value < 0.005 vs CTR, * *p*-value < 0.05 vs IL-1α. CTR negative control. **B** Analysis of antioxidant defense activity on OA hACs transfected with single hsa-miR17- 5p, hsa-miR20-5p, hsa-miR21-5p, hsa-miR29a- 3p, hsa-miR29b-3p, hsa-miR29c-3p, or with scramble control (100 nM). Data are represented as mean ± SD for the following oxidative damage accumulation markers: GR Catalase, GSH, and GSSG (*n* = 3). One-way ANOVA, # *p*-value < 0.05 vs CTR, * *p*-value < 0.05 vs IL-1α. CTR negative control. 4-HNE 4-hydroxynonenal, 8-OHdG 8-hydroxy-2′-deoxyguanosine, GR glutathione reductase, GSH reduced glutathione, GSSG oxidized glutathione, MDA malondialdehyde, ROS reactive oxygen species. **C** Representative western blot of NRF2, KEAP1 and Actin expression on OA hACs transfected with 100 nM hsa-miR17-5p, hsa-miR20 - 5p, hsa-miR21-5p, hsa-miR29a-3p, hsa-miR29b-3p, hsa-miR29c-3p, or with scramble control. **D** Analysis of NRF2/KEAP1 expression quantification on western blots of OA hACs transfected with single hsa-miR17- 5p, hsa-miR20-5p, hsa-miR21-5p, hsa-miR29a- 3p, hsa-miR29b-3p, hsa-miR29c-3p, or with scramble control (100 nM). Data are represented as mean ± SD (*n* = 3). One-way ANOVA, # *p*-value < 0.005 vs CTR, * *p*-value < 0.05 vs IL-1α. **E** Flow cytometry quantification of intracellular ROS in OA hACs transfected with 100 nM hsa-miR17-5p, hsa-miR20- 5p, hsa-miR21-5p, hsa-miR29a-3p, hsa-miR29b-3p, hsa-miR29c-3p or with scramble control. Data are represented as Fold change of DCF mean fluorescence intensity with respect to CTR. (*n* = 4), One-way ANOVA, # *p*-value < 0.005 vs CTR, * *p*-value < 0.05 vs IL-1α.

To investigate whether the iEVs can modulate redox status and oxidative damage through miRNA-mediated signaling, intracellular ROS levels, oxidative damage markers, and antioxidant response were evaluated on chondrocytes transfected by the selected miRNA. All miRNA transfections led to a reduction in markers of oxidative damage, as evidenced by decreased levels of MDA, 4-HNE, 8-OHdG, and nitrotyrosine (Fig. 6A). Notably, the reduction in oxidative damage was more pronounced in chondrocytes transfected with three miR-29 family members. These findings were aligned by an increase in the activity of key antioxidant enzymes, including catalase and GR activity in transfected cells (Fig. 6B), which supported the antioxidant defense system as shown by the increment of GSH level and the concomitantly decrement of GSSG content (Fig. 6B).

To investigate the activation of upstream antioxidant defense pathways, we also analyzed NRF2 (Nuclear factor erythroid 2_related factor), and KEAP1 (Kelch-like ECH-associated protein1) proteins expression by Western blot (Fig. 6C). The NRF2/KEAP1 ratio exhibited a heterogeneous increase following treatment with the different miRNAs (Fig. 6D and Supplementary Fig. 4A, B), indicating that miRNA administration to inflamed hACs is able to activate the antioxidant response.

Finally, we have evaluated intracellular ROS level after either the administration of inflammatory stimuli or miRNAs transfection. As expected, IL-1α primed hACs exhibited a significant increase in ROS levels compared to the control condition (Fig. 6E). Conversely, transfection with three miR-29 family members (i.e., hsa-miR29a-3p, hsa-miR29b-3p, and hsa-miR29c-3p) significantly reduced intracellular ROS levels. In contrast transfections with hsa-miR17-5p, hsa-miR20-5p, and hsa-miR21-5p did not lead to a statistically significant reduction in oxidative stress (Fig. 6E), although a decreasing trend was observed.

Taken together, these findings highlight the therapeutic potential of miRNAs in mitigating oxidative stress associated with OA by enhancing antioxidant enzymes activity and overall antioxidant capacity. Notably, the anti-inflammatory and redox-regulatory effects are not mediated by a single miRNA, but rather by a group of EV-associated miRNAs acting synergistically through the modulation of multiple molecular pathways.

### miRNAs pool shows a synergic effect in regulating inflammation and oxidative stress

To assess whether the beneficial effects observed following iEV treatment, partially mimicked by single miRNA transfections, could be attributed to synergic miRNA activity, we performed a series of analyses replicating our established readouts. We compared the effects of SD1-iEVs with those of a miRNA pool (as detailed in the “Materials and methods”) in primary hACs stimulated with IL-1α, including a scramble control in all experiments.

**Fig. 7:**
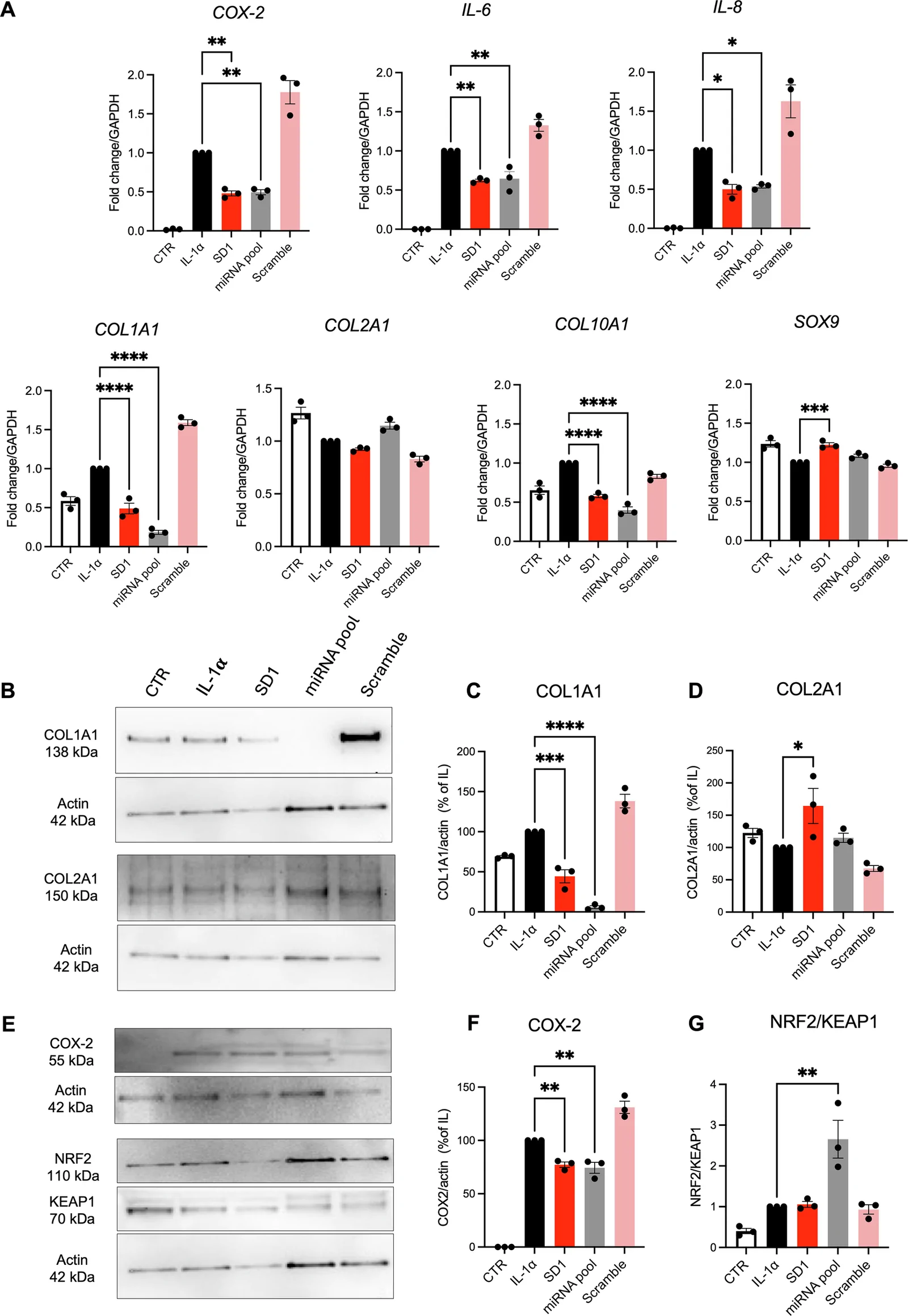
miRNAs pool shows synergic anti-inflammatory effects and regulates redox balance in chondrocytes. **A** Human articular chondrocytes were transfected with SD1 iEVs, six miRNAs pool or with scramble control. Graphs show the relative expression of target genes *IL-6, IL-8, and COX-2, COL1A1, COL2A1, COL10A1* and *SOX9* analyzed by quantitative real-time qPCR. Data are shown as mean ± SD, (*n* = 3). One-way ANOVA, * *p*-value < 0.05, ** *p*-value < 0.01, *** *p*-value < 0.001, **** *p*-value < 0.0001. CTR negative control. **B** Representative western blot of COL1A1, COL2A1 and Actin expression on OA hACs transfected with SD1 iEVs, 100 nM miRNAs pool or with scramble control. **C** Quantification of COL1A1 protein expression in Western blot of OA hACs transfected with SD1 iEVs, 100 nM miRNAs pool or with scramble control. Data are shown as mean ± SD, (*n* = 3). One-way ANOVA, *** *p*-value < 0.001, **** *p*-value < 0.0001. CTR negative control. **D** Quantification of COL2A1 protein expression in Western blot of OA hACs transfected with SD1 iEVs, 100 nM miRNAs pool or with scramble control. Data are shown as mean ± SD, (*n* = 3). One-way ANOVA, * *p*-value < 0.05. CTR negative control. **E** Representative western blot of COX-2, NRF2, KEAP1, and Actin expression on OA hACs transfected with SD1 iEVs, 100 nM miRNAs pool or with scramble control. **F** Quantification of COX-2 protein expression in Western blot of OA hACs transfected with SD1 iEVs, 100 nM miRNAs pool or with scramble control. Data are shown as mean ± SD, (*n* = 3). One-way ANOVA, ** *p*-value < 0.01. CTR negative control. **G** Quantification of NRF2/KEAP1 protein expression in Western blot of OA hACs transfected with SD1 iEVs, 100 nM miRNAs pool or with scramble control. Data are shown as mean ± SD, (*n* = 3). One-way ANOVA, ** *p*-value < 0.01. CTR negative control.

As shown in Fig. 7A, the miRNA pool was as effective as SD1-iEVs in reducing the gene expression of *COX-2*, *IL-6*, and *IL-8*, all of which were significantly upregulated by the pro-inflammatory stimulus. Regarding chondrogenic markers, the miRNA pool exerted an even more pronounced inhibitory effect on *COL1A1* and *COL10A1* expression compared to the already significant reduction induced by SD1-iEVs. In contrast, the miRNA pool did not significant modulate *COL2A1* or *SOX9* transcript levels (Fig. 7A).

To corroborate these findings at the protein level, we also performed western blot analyses and immunofluorescence staining for COL1A1 and COL2A1. We observed a significant decrease in COL1A1 protein levels following SD1-iEV treatment, which was further intensified by the miRNA pool (Fig. 7B, C and Supplementary Fig. 5A, B). Expression of collagen isoform COL2A1 was increased by SD1-iEVs but remained unchanged after exposure to the miRNA pool (Fig. 7B, D and Supplementary Fig. 6A, B). Regarding the inflammatory response, both treatments significantly attenuated COX-2 protein expression after inflammatory stimuli administration (Fig. 7E, F and Supplementary Fig. 7A, B).

Finally, we evaluated the antioxidant response by analyzing the expression of NRF2, a key regulator of cellular antioxidant defenses, and its negative regulator KEAP1. Notably, the NRF2/KEAP1 ratio was significantly increased following treatment with the miRNA pool (Fig. 7E, G and Supplementary Fig. 4C, D), supporting the robust activation of the antioxidant defense system observed.

## Discussion

Osteoarthritis (OA) affects millions of people globally and is primarily treated with NSAIDS or steroids that offer only symptomatic relief. The lack of curative therapy has driven interest in biological approaches, particularly MSC and their extracellular vesicles, due their anti-inflammatory potential. Preclinical studies show that EVs, from human adipose-derived MSCs reduce inflammatory mediator production, and their therapeutic potential in OA is supported by an ongoing clinical trial evaluating adipose- derived stem cell secretome on human osteochondral explants (ClinicalTrials.gov ID NCT04223622).

Other studies report that bone marrow-derived MSC-EVs suppress inflammatory mediators (COX-2, IL-1α/β, IL-6, IL-8, IL-17), reduce TNF-α–induced inflammation, inhibit NF-κB nuclear translocation, and promote proteoglycan deposition and collagen II production [39]. However, affinity issues, donor-dependent heterogeneity, and early senescence in primary cultures limit the translational potential of MSC-based therapies [40]. Moreover, we previously showed that MSC-derived EVs display strong anti-inflammatory effects at early culture passages, which decline with prolonged culture, indicating that EVs biological potency is influenced by MSC aging [41]. To overcome this limitation, we are exploring alternative MSC sources and their corresponding EVs to preserve or enhance therapeutic efficacy.

In this context, MSCs derived from human iPSCs have emerged as a promising source to produce EVs and we hypothesized that they could overcome previous current challenges [42]. Furthermore, recent studies have shown that EVs secreted by both human bone marrow-derived MSCs and iMSCs exhibit comparable immunomodulatory effects in vitro, as demonstrated by a multi-donor mixed lymphocyte reaction assay [43].

Based on these findings, we recently demonstrated the anti-inflammatory potential of iEVs in OA treatment, but observed notable batch-to-batch variability, likely due to heterogeneity among parental iMSCs populations, warranting further investigation [41].

In this study, three previously characterized iPSC-derived MSC lines (SD1, SD2, and SD3) were long-term cultured, and EVs were collected at early, mid, and late passage to identify the optimal harvest window based on yield and phenotypic characteristics [30]. iMSCs exhibited low senescence at P12, except for SD2.

Interestingly, CD44, a hyaluronic acid receptor, showed increased expression over time, suggesting potential chondrogenic differentiation at late passages of culture [44]. Moreover, EV secretion increased from P8 to P16, but iEVs from later passages lost specific markers, likely due to senescence, and their miRNA cargo became undetectable, suggesting impaired functionality [45].

P12 iMSC in exponential growth, maintained typical fibroblastic morphology and expressed mesenchymal markers (CD44, CD90, CD105) in over 98% of cells [46], making this stage optimal for iEV isolation. All three iMSC lines retained these phenotypical features long-term, highlighting iMSC culture heterogeneity, a potential limitation for therapeutic use.

Focusing on P12-derived iEVs, we evaluated anti-inflammatory and pro-chondrogenic effects, as well as redox modulation and miRNA cargo delivery in an IL-1α primed hAC in vitro OA model [47]. Although several alternative models exist to study inflammatory signaling, IL-1α stimulation was selected in this study due to its ability to induce a rapid, robust, and well-characterized activation of NF-κB, allowing controlled investigation of downstream molecular mechanisms [27]. Future studies should extend these findings by incorporating additional inflammatory stimuli, such as TNF-α or IL-1β, as well as models based on NF-κB pathway modulation. Moreover, integrating mechanical or compressive stress will be essential to better define iEVs efficacy [48].

SD1 and SD3-iEVs reduced IL-6 and IL8 expression and inhibited NF-κB nuclear translocation, whereas SD2-iEVs had minimal effects, indicating SD2 may be a suboptimal iEVs source. Our findings suggest that iEVs progressively lose their phenotypic identity as donor cells undergo senescence. Consistent with recent published studies [41], we observed heterogeneity among iMSC lines [49], including a potential premature onset of senescence. Interestingly, in the SD2 iMSC line, early senescence is associated with a progressive loss of iEV phenotype, occurring independently of particle secretion rates, and potentially leading to impaired anti-inflammatory activity. Furthermore, iEVs supported pro-chondrogenic marker expression, particularly SD3-derived EVs, which significantly increased SOX-9 and COL2A1 expression.

Furthermore, the redox potential of iEVs was evaluated on inflamed hACs, as oxidative stress is a key contributor of OA pathogenesis. In aging and OA cartilage, disrupted redox signaling shifts the balance toward catabolic process, promoting matrix degradation, with elevated ROS consistently reported in patients [50].

iEVs treatment restored redox balance by reducing oxidative damage and modulating ROS-detoxifying enzyme activity. MSC-EV are known to have limited capacity to restore redox homeostasis [51], confirming a different cargo for iMSC-derived EVs with respect to MSC-derived EVs.

To investigate this mechanism, we analyzed iEVs miRNA cargo. Late-passage (P16) iEVs, corresponding to higher senescence levels, lacked detectable miRNAs, in line with previous findings indicating that senescence is associated with alterations in extracellular vesicle cargo and reduced functional activity [52, 53]. Similarly, P12 SD2-iMSC, which showed premature senescence, produced iEVs devoid of miRNAs. In contrast, P12 iEVs from other lines contained miRNAs targeting pathways implicated in OA, including differentiation, inflammation, and angiogenesis, consistent with their protective effects in an in vitro OA model. Both P8 and P12 iEVs contain miRNAs targeting key genes involved in the cellular senescence pathway, possibly protecting iMSCs from premature senescence onset.

Previously, we showed that MSC-EV miRNAs, such as miR-145 and miR-214, protect chondrocytes from IL-1α–induced pro-inflammatory cytokine expression (IL-6, IL-8, COX-2) [27]. These miRNAs were absent in iEVs, consistent with the weaker anti-inflammatory effects observed. Among iEVs-enriched miRNAs, only miR-20-5p, involved in chondrogenic differentiation and cartilage repair, reduced COX-2 and IL-8 but not IL-6, highlighting the limited anti-inflammatory potential of iEVs compared to MSC-EVs [54].

Notably, members of the miR29-family (miR-29a-3p, miR-29b-3p, miR-29c-3p), which regulate cartilage homeostasis and OA progression, decreased IL-6 levels in OA in vitro model, without affecting IL-8 or COX-2 [55].

The treatment with iEVs has also been able to modulate the redox state of chondrocytes, acting both on the production of oxidative stress and the accumulation of related damage, as well as on the activation of antioxidant defenses. Specifically, the treatment with the EVs counteracts the oxidative damage caused by IL-1α by both inducing a lower production of ROS and promoting the enhancement of antioxidant protection, ultimately resulting in a reduction of oxidative damage to lipids, proteins, and nucleic acids. Our data also suggest that these effects are mediated by specific miRNA. In addition, the observed increase in the NRF2/KEAP1 ratio supports the ability of both several single miRNA and miRNA pool transfection to enhance the expression of antioxidant defenses [56]. While the miRNA pool treatment did not alter KEAP1 expression levels, it significantly increased NRF2 expression, resulting in a higher NRF2/KEAP1 ratio, which is indicative of activate antioxidant and cytoprotective activity.

Our findings suggest that single miRNA administration has heterogenous effects, implying that these miRNAs likely act synergistically to strongly modulate inflammation and redox balance in an in vitro OA model [57]. Indeed, the miRNA pool more effectively reduced the expression of key pro-inflammatory markers (COX-2, IL-6, and IL-8) at both transcript and protein levels. Interestingly was also observed a pronounced anti-fibrotic effect of the miRNA pool, largely mediated by the miR-29 family, which is known to target COL1A1, suppressing its expression [58, 59]. This finding highlights a potential mechanism through which miRNAs can mitigate fibrotic remodeling in OA. Finally, the miRNA pool robustly enhances antioxidant defenses, indicating that the synergistic action of multiple miRNAs ameliorates the restoration of cellular redox homeostasis. Taken together, these results highlight the synergistic activity of selected miRNAs and support their therapeutic potential for modulating multiple pathogenic processes in OA.

Overall, our results support iMSCs as a viable alternative to primary MSCs for EV production. iMSCs maintain stable long-term cultures and secrete higher levels of iEVs, which retain anti-inflammatory activity and redox-modulating potential through mid-passages in an in vitro OA model [60, 61].

The miRNA cargo is influenced by culture passage, with P12 iEVs containing molecules targeting genes involved in OA and cartilage homeostasis, playing a key role in mediating iEVs effects. Among the six identified miRNAs, most modulate pro-inflammatory and pro-chondrogenic markers heterogeneously, whereas the miR-29 family shows a more consistent role in restoring antioxidant defenses and controlling ROS accumulation.

In conclusion, iMSCs-derived EVs positively modulate the inflammation state and enhance antioxidant defenses, reducing ROS accumulation induced by IL-1α in the OA in vitro model used. The non-homogeneous and milder effects observed following miRNA transfection prompt that transfection with a single miRNA may be insufficient to reach the stronger iEVs treatment effects. Our data indicate that the enhanced anti-inflammatory, anti-fibrotic, and antioxidant effects of iEVs are largely mediated by the synergistic action of the selected miRNAs, highlighting the therapeutic potential of a miRNA-based combinatorial approach for OA. This results in OA pathophysiology might be attributable to a synergic action of various molecules present in the iEVs cargo, suggesting that a synergistic miRNA-based approach could lead to improved therapeutic outcomes [57].

Although all these findings were demonstrated in an in vitro model, the iEVs were simultaneously administrated in a OA murine model, yielding encouraging results. iEVs treatment promotes cartilage repair while inhibiting cartilage degradation, as illustrated by increased COL2 and reduced Mmp13 expression in murine model of OA [62].

## Materials and methods

### Generation of multiple iMSC lines from a single iPSC line

The iPSC line ChiPS 22 (Takara Bio, Sweden, European Stem Cell Registry no. CEBi001-A) was plated and differentiate as previously reported [41]. Cells were routinely tested for mycoplasma contamination and karyotype stability. In passages 4–5, cells had a fibroblastic-like appearance and were further characterized for MSC features by surface marker expression analysis and trilineage differentiation. Three different batches of iMSCs were generated from the same iPSC line named SD1, SD2, and SD3.

### Cell culture

SD1, SD2 and SD3 iMSCs were cultured in MEM Alpha Medium (1X) + GlutaMAX (GiBCo, ref. 32561-029), supplemented with a mixture of 100 U/mL penicillin and 100 mg/mL streptomycin (Euroclone, ref. ECB3001D), 1 μl/ml ITS Premix (CORNING ref. 354350), 5 ng/ml transforming growth factor beta 1 (TGF-β) and 5 ng/mL fibroblast growth factor 2 (PrepoTech) (complete medium), along with the Xeno-free Purstem supplement to avoid the presence of serum in culture medium. Cells were maintained at 37 °C and 5% CO_2_. At 90% confluence, cells were detached with TrypLE Express (Gibco ref. 12604-013) and used for further experiments. SD1, SD2, and SD3 cells were collected at three different passages (P): P8, P12, and P16 and used for the subsequent analyses.

Human articular chondrocytes (hACs) were isolated from the femoral head or femoral condyles of patients undergoing total hip or total knee arthroplasty as previously described [63]. Informed consent was obtained from each patient, and the study received approval from the Regional Ethical Committee (CER Liguria: 372/2019 Genoa, Italy). The donor cohort was sex-balanced with a mean age of 62 ± 7 years.

### Growth kinetics

The growth kinetics of iMSCs were analyzed from passage P8 to P16. At each passage, cells were seeded at a density of 7 × 10³ cells/cm². Continuous culture was maintained until the cells reached confluency. Cells were then harvested using TrypLE Express and counted manually using a Neubauer counting chamber. Dead cells were stained with Trypan Blue to ensure accurate quantification. Population doubling time (PDT) was calculated using the following formula:

$$n^{\circ} \,{of}\,{doublings}={\log }_{2}\frac{{counted}\,{cells}}{{seeded}\,{cells}}$$

### Cell senescence evaluation: senescence-associated β-galactosidase activity

β-galactosidase staining was performed to assess senescence levels in iMSCs at P8, P12, and P16, in accordance with the manufacturer’s instructions of the Senescence β-Galactosidase Staining Kit (Cell Signaling Technology ref. #9860). Images were acquired with a Leica-DM1 microscope, and the subsequent analysis was performed using ImageJ software.

### iMSC phenotypic characterization

The iMSCs surface phenotype was analyzed by flow cytometry. Briefly, 50,000 iMSCs/tube for P8 and P16 were incubated for 30 min at room temperature in the dark with specific conjugated antibodies: FITC-anti-human CD31, FITC-anti-human CD34, PE-anti-human CD90 and PE-anti-human CD105 (BD Pharmingen, San Diego, CA, United States). Likewise, cells were stained with the corresponding IgG isotype conjugated with FITC (BD Bioscience) or PE (BD Pharmingen). Samples were run on a CytoFLEX Flow Cytometer (Beckman Coulter, Brea, CA, United States), and data were analyzed using FlowJo software (BD Biosciences).

### Separation of EVs from iMSCs

The iMSCs were continuously cultured in a xeno-free medium. Conditioned medium (CM) from cells was collected at three passages and immediately centrifuged at 300 × *g* for 10 min at 4 °C to remove dead cells and debris. Subsequently, the supernatant was centrifuged at 2000 × *g* for 20 min at 4 °C to eliminate apoptotic bodies and concentrated using Amicon Ultra Filters (molecular weight cut-off of 3 kDa; Millipore, ref. UFC9003).

EVs were separated by a differential ultracentrifugation protocol [41].

The concentration of membrane-bound proteins on the surface of freshly isolated EVs was measured using the BiCinchoninic Acid (BCA) assay (Thermo Scientific Pierce, Rockford, IL, USA) following the manufacturer’s instructions.

### Nanoparticle tracking analysis (NTA)

EVs were characterized and quantified using Zeta View (Particle Metrix GmbH, Germany) NTA equipped with two lasers (488 nm and 640 nm). Zeta View 8.05.14_SP7 software was used. After calibration with 100 nm polystyrene beads, samples diluted in 0.22-μm-filtered PBS 1X (1:100) were injected using a 1 ml syringe. Size distribution analyses of 11 different positions were performed for each sample on at least three different EV preparations. The particle number was then normalized to the number of seeded cells.

### Western blot

iEV proteins were isolated by RIPA buffer supplemented with protease inhibitors (Cell Signaling Technology, Danvers, MA, United States) and quantified by the BCA assay; one μg of proteins was run into 4–12% Tris-Glycine gels (Invitrogen). After blocking with 5% non-fat dry milk in TTBS (500 mM NaCl, 0.1% v/v Tween, and 20 mM Tris, pH 7.5) for 1 h at room temperature, membranes were incubated overnight at 4 °C with specific primary antibodies diluted in 2.5% non-fat dry milk in TTBS and directed against syntenin-1 (dilution 1:1000, ab133267, Abcam), flotillin-1 (dilution 1:10,000, ab133497, Abcam, Cambridge, United Kingdom), CD63 (dilution 1:500, 10628D, Invitrogen, Waltham, MA, United States), CD81 (dilution 1:1000, 555675, BD Biosciences). Membranes were incubated with specific HRP-conjugated goat anti-rabbit/mouse antibodies diluted in 2.5% non-fat dry milk in TTBS (dilution 1:2000, Cell Signaling Technology, Danvers, MA, United States) for 1 h at room temperature. Proteins were detected and analyzed for optical density using an enhanced chemiluminescence system (Alliance 6.7 WL 20 M, UVITEC, Cambridge, UK) and UV1D software (UVITEC) or developed on radiographic film.

hACs, after either iEVs or miRNA treatment, were lysed in 7 M urea, 4 M thiourea, and 1% CHAPS supplemented with protease inhibitors (Cell Signaling Technology, Danvers, MA, USA). Protein concentration was determined using the Bradford assay. Fifteen micrograms of total protein were separated on 4-12% Tris-Glycine gels (Invitrogen) and transferred onto nitrocellulose membranes. The following primary antibodies were used: NRF2 (1:1000, AB137550, Abcam, Cambridge, UK), KEAP1 (1:1000, 8047S, Cell Signaling Technology), COX-2 (1:1000, 4842S, Cell Signaling Technology), COL1A1 (1:1000, SP1.D8, Developmental Studies Hybridoma Bank), COL2A1 (1:1000, C2C21, Developmental Studies Hybridoma Bank), and Actin (1:5000, A2066, Sigma-Aldrich). Membranes were then incubated with HRP-conjugated goat anti-rabbit or anti-mouse secondary antibodies diluted in 2.5% non-fat dry milk in TTBS (1:2000, Cell Signaling Technology) for 1 h at room temperature. Protein bands were detected using an enhanced chemiluminescence system (Alliance 6.7 WL 20 M, UVITEC, Cambridge, UK). Densitometric analysis was performed using UV1D software (UVITEC) and quantified with ImageJ.

### Non-conventional flow cytometry analysis of EVs

EVs were suspended in 0.22-μm-filtered PBS/EDTA, distributed in flow cytometry tubes (1 × 10^8^ EVs/tube), and stained with 1 μM CFDA-SE (VybrantTM CFDA SE Cell Tracer Kit, Thermo Fisher Scientific) at room temperature to visualize intact particles. Within the CFDA-SE positive events EVs expression of CD9 (APC Mouse Anti-Human CD9, ref. 312108, BioLegend, San Diego, CA, United States), CD63 (PE-Cy7 Mouse Anti-Human CD63, ref. 561982, BD Biosciences), and CD81 (BV421 Mouse Anti-Human CD81, ref. 740079, BD Biosciences), was evaluated. CytoFLEX flow cytometer (Beckman Coulter) was used for experiments and data were analyzed using FlowJo. Dimensional analysis was performed according to Gorgun et al. [64].

### Chondrocytes EVs treatment

Confluent hACs (P2) were treated for 16 h with 5 ng/µL (corresponding to 200 U/mL) interleukin-1 alpha (IL-1α) ± 1 μg/mL of iEVs. Negative controls without IL-1α and EVs and positive controls treated only with 200 U/mL IL-1α were also included [63].

### MiRNA profiling by microarray

Total EV-RNA was isolated by miRNeasy Micro Kit (Qiagen). MiRNA content was evaluated by the Qubit® 2.0 Fluorometer using RNA High Sensitivity kit (Thermo Fisher Scientific). The expression levels of 2549 EV-miRNAs were profiled by microarray using the SurePrint Human miRNA platform (Agilent Technologies; AMADID: 070156). Eighty ng of EV-RNA and Spike-in controls were labeled according to the protocol and hybridized. Fluorescent signals were then acquired by a G2565CA scanner (Agilent Technologies), and data were extracted by Feature Extraction software v.9.5.3.1 (Agilent Technologies). Raw data were processed using the limma R package for microarray analysis. Background correction and between-array normalization were carried out using the *normexp* method, with an offset = 20, and the *quantile* method, respectively. Class comparison between treatment and control groups was performed by fitting linear models to expression data and then applying an empirical Bayes method to borrow strength across miRNAs and identify significant differences. MiRNAs with *p* < 0.05 (−log10 = 1.3) and log (fold change) > 0.58 (or <−0.58) were assumed to represent upregulated (or downregulated) miRNAs, respectively. The list of most significant upregulated and downregulated EV-miRNAs was uploaded on DIANA miRPath 3.0. Pathway involvement was first investigated by the KEGG (Kyoto Encyclopedia of Genes and Genomes) pathway database. Target genes were predicted in silico by using miRsystem.

### Transfection of chondrocytes with double-stranded (ds) miRNA mimic

hACs were seeded in 24-well plates (2 × 10⁵ cells/well) and cultured for 24 h prior to transfection. Cells were transfected with the following mirVana miRNA mimics: hsa-miR-17-5p, hsa-miR-20-5p, hsa-miR-21-5p, hsa-miR-29a-3p, hsa-miR-29b-3p, and hsa-miR-29c-3p, either individually (100 nM each) or as a combined miRNA pool. For pooled transfections, the total miRNA concentration was maintained at 100 nM, with each individual miRNA present at an equimolar fraction of the total concentration. A negative control (Scramble) was also included in all experiments (100 nM, Thermo Fisher Scientific). Transfection was performed using Lipofectamine 3000 (Invitrogen) in Optimem medium (GiBCo) and according to the protocol. Scramble-transfected cells were used as controls in all experiments. Transfection efficiency was checked after 48 h, evaluating the miRNA expression by qPCR. Concomitantly, expression of miRNA target genes (i.e., *IL-6, IL-8*, and *COX-2, COL1A1, COL2A1, COL10A1*, and *SOX-9)* was assessed by qPCR. Briefly, after 48 h of miRNA transfection in the presence of 200 U/mL IL-1α, the expression of total RNA from miRNA-transfected chondrocytes and control-matched cells was isolated and the relative expression was evaluated using specific primers provided in Table 5. In miRNA-transfected samples treated with IL-1α, the oxidative stress and damage markers as well as the antioxidant response have also been evaluated.

**Table 5:** Primer used for quantitative real-time PCR.

	Forward primer	Reverse primer
*GAPDH*	5’-CCATCTTCCAGGAGCGAGAT-3’	5’-CTGCTTCACCACCTTCTTGAT-3’
*COX-2*	5’-AATTGCTGGCAGGGTTGCT-5’	5’-GGTCAATGGAAGCCTGTGATACT-3’
*IL-6*	5’-TGACGACCTAAGCTGCACTT-3’	5’-GGGCTGATTGGAAACCTTATTA-3’
*IL-8*	5’-CGTGGCTCTCTTGGCAGC-3’	5’-TTAGCACTCCTTGGCAAAACTG-3’
*COL1A1*	5’-CAGCCGCTTCACCTACAGC-3’	5’-TTTTGTATTCAATCACTGTCTTGCC-3’
*COL2A1*	5’-GGCAATAGCAGGTTCACGTACA-3’	5’-CGATAACAGTCTTGCCCCACTT-3’
*COL10A1*	5’-TGGGTAGGCCTGTATAAGAACGG-3’	5’-CATGGGAGCCACTAGGAATCCTGAGA-3’
*SOX-9*	5’-CCCGCACTTGCACAACG-3’	5’-TCCACGAAGGGCCGCT-3’

### Quantitative real-time PCR (qPCR)

Following iEV treatments or miRNA transfection, RNA of cells was isolated by TRIzol™(Thermo Fisher Scientific, ref. 15596018), and quantified by the BioSpectrometer (Eppendorf, Hamburg, Germany). Two μg of total RNA were retrotranscribed using SuperScript™ IV VILO™ Master Mix (Thermo Fisher Scientific, ref. 11756050). Transcript levels of interleukin-6 (*IL-6*), interleukin-8 (*IL-8*), ciclooxygenase-2 (*COX-2*), collagen-1 (*COL1A1*), collagen-2 (*COL2A1*), collagen-X (*COL10A1*), SRY-box transcription factor-9 (*SOX-9*), and genes were evaluated by qRT-PCR by 7500 Fast Real-Time PCR System (Applied Biosystems, Foster City, CA, USA). The selected human-specific primer sequences are provided in Table 5. *GAPDH* was used as an endogenous control for normalization. Data were expressed as fold changes relative to *GAPDH* using the 2-ΔΔCt formula.

### NF-κB nuclear translocation

Four different pools of hACs were cultured in 24-well plates on glass coverslips (20,000 cells) and treated for 16 h with 100 U/mL IL-1α and 1 μg/mL EVs. Cells were fixed in 3.7% PFA and permeabilized with 0.5% Triton X-100 in PBS. Non-specific binding was prevented by incubation with 20% goat serum (Euroclone) in PBS for 30 min at 4 °C. Samples were incubated with rabbit anti-NF-κB p65 antibody (1:400; Cell Signaling, Danvers, MA, USA) overnight at 4 °C, followed by goat anti-rabbit Alexa fluor-488 (Life Technologies, Grand Island, NY, United States) for 30 min at 4 °C and nuclei were counterstained with DAPI. Data were acquired Apotome microscope (Carl Zeiss) and analyzed using imageJ software. For each sample, five different regions of interest were acquired for the subsequent analysis.

### Intracellular ROS evaluation

Intracellular ROS levels of hACs after iEVs or miRNA treatment were measured using the 2′,7′-dichlorodihydrofluorescein diacetate (H2DCFDA, Thermo Fisher Scientific, Waltham, MA, USA) staining. Briefly, cells were incubated with H2DCFDA at 10 µM for 15 min at 37 °C, washed three times, and trypsinized. Samples were measured on a FACSCalibur flow cytometer (BD Biosciences, San Diego, CA) and the mean fluorescence value for each cell population was estimated. The analysis was confined to viable cells only, after gating based on forward- and side-scatter characteristics.

### Oxidative damage accumulation assay

To evaluate oxidative damage accumulation in hACs after iEVs or miRNA treatment, the levels of malondialdehyde (MDA) and 4-hydroxynonenal (4-HNE), 8-hydroxy-2′-deoxyguanosine (8-OHdG), and nitrotyrosine were measured as lipid peroxidation, oxidative DNA damage, and protein oxidation markers, respectively. Intracellular levels of 4-HNE, 8-OHdG, and nitrotyrosine were assessed using the Lipid Peroxidation (4-HNE) Assay Kit (code: ab287803), 8-hydroxy-2′-deoxyguanosine ELISA Kit (code: ab201734), and Nitrotyrosine ELISA Kit (code: ab116691), respectively, all provided by Abcam. The protocols were performed according to those reported in Cossu et al. [65]. The analyses were performed according to the manufacturer’s protocols, with 50 μg of total protein used for each assay.

### Assay of intracellular GSH and GSSG levels

The intracellular concentrations of reduced glutathione (GSH) and oxidized glutathione (GSSG) were determined using the Glutathione GSH/GSSG Assay Kit (code: MAK440), both supplied by Merck (Darmstadt, Germany). The assays were performed following the instructions provided by the manufacturer, using 50 μg of total protein per sample.

### Glutathione reductase and Catalase activity assay

A total of 50 μg of total protein was used for both antioxidant enzyme activity assays, following the protocol reported in Cossu et al. [65].

### Immunofluorescence staining

hACs were seeded at 50,000 cells on 18 mm glass coverslips and treated with 200 U/mL IL-1α in the presence of SD1-iEVs or a miRNA pool. Fixation, permeabilization, and blocking were performed as described in the NF-κB Nuclear Translocation section. Cells were incubated overnight at 4 °C with primary antibodies diluted in blocking solution: COX-2 (1:100, 4842S, Cell Signaling Technology), COL1A1 (1:100, SP1.D8, DSHB), COL2A1 (1:100, C2C21, DSHB), CD44 (1:100, ab189524, Abcam), and β-tubulin (1:1000, Sigma-Aldrich). Alexa Fluor 488- or 568-conjugated secondary antibodies (1:500, Life Technologies) were applied for 2 h at room temperature, and nuclei were counterstained with DAPI. Images were acquired using a Nikon A1 confocal microscope.

Quantitative image analysis was performed using ImageJ. For COL1A1 and COX-2, the number of positive cells was quantified, while COL2A1 expression was assessed by total cellular fluorescence intensity. Nuclei were segmented from DAPI maximum intensity projections after background subtraction (rolling ball radius: 40 pixels), Otsu thresholding, and watershed separation. Objects smaller than 50 pixels or with circularity <0.3 were excluded. Nuclear ROIs were expanded by 5 µm to approximate cell area. Background fluorescence was estimated from regions outside ROIs using an inverted cell mask and subtracted from measurements. Fluorescence intensity per ROI was then extracted and normalized.

### Statistical analysis

Data were analyzed using GraphPad Prism 9.3 software (GraphPad Software, Inc., San Diego, CA, USA). Statistical comparisons among groups were conducted using one-way analysis of variance (ANOVA), followed by Tukey’s post hoc multiple comparison test. Levels of significance were set at *p* < 0.05 (*p < 0.05, **p < 0.01, ***p < 0.001, ****p < 0.0001 and #*p* < 0.0005). Given the technical nature of the assays and the variability observed in iMSC lines, three independent biological replicates were considered sufficient to ensure adequate precision in detecting the effect size. In contrast, due to the higher intrinsic variability of primary human chondrocytes, at least four independent biological replicates were included. Figure legends report independent experiment numbers for each analysis performed. Data are shown as mean ± SD.

## Supplementary information

Figure S1

Figure S2.

Figure S3.

Figure S4.

Figure S5.

Figure S6.

Figure S7.

Supplementary Legend

Original Data

## Data Availability

All relevant data supporting the findings of this study are available within the paper, its supplementary information, and from the corresponding authors upon reasonable request. Raw data were uploaded to the Gene Expression Omnibus repository GSE309880.
