# Supplementary material for: iMSC-derived extracellular vesicles and their miRNA cargo influence inflammation and oxidative damage in an in vitro osteoarthritis model
Source: Cell Death Dis. 2026 Jun 24;17(1):781. doi: 10.1038/s41419-026-09033-0 (PMC13547349; doi:10.1038/s41419-026-09033-0)
Supplement: Supplementary file 1 [file 41419_2026_9033_MOESM9_ESM.docx]

CD63

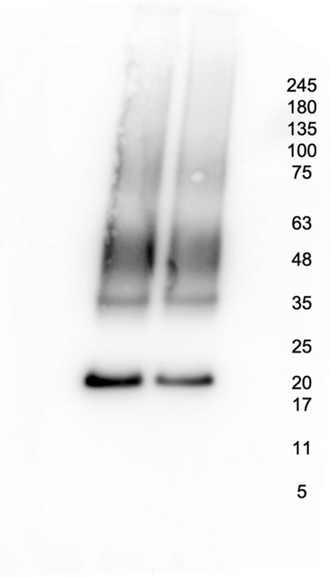

CD81

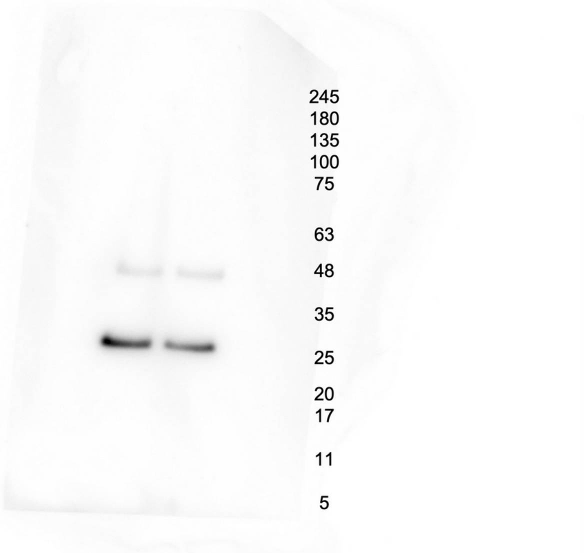

Flotillin-1

Syntenin-1

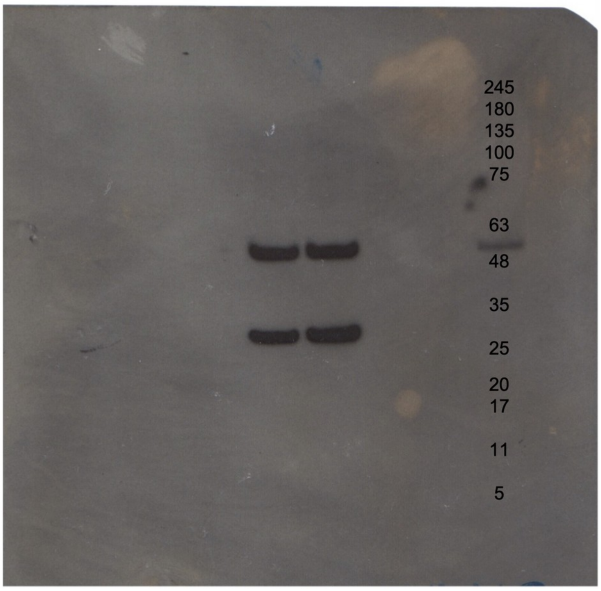

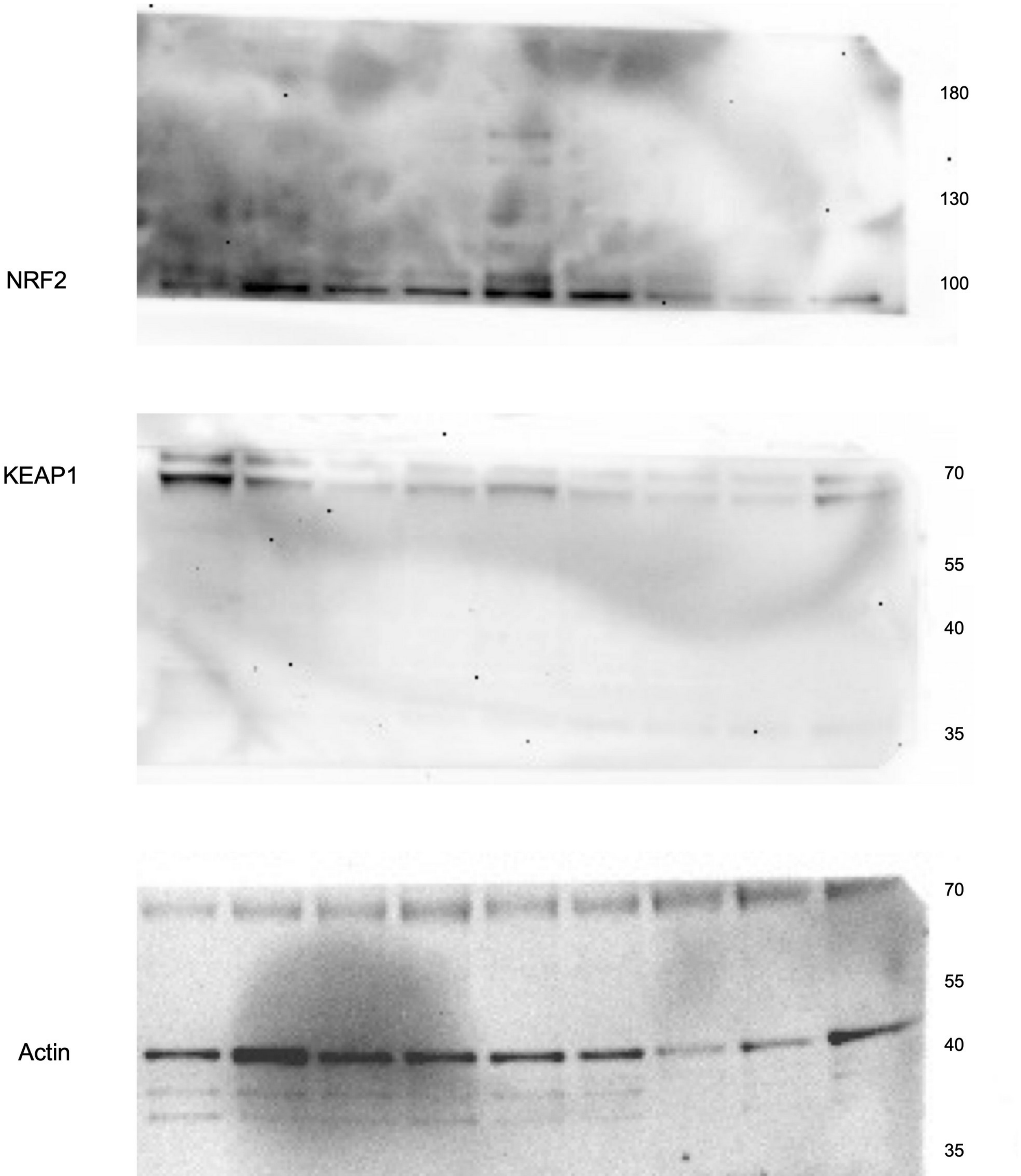
NRF2
180
130
100
KEAP1
70
55
40
35
Actin
70
55
40
35

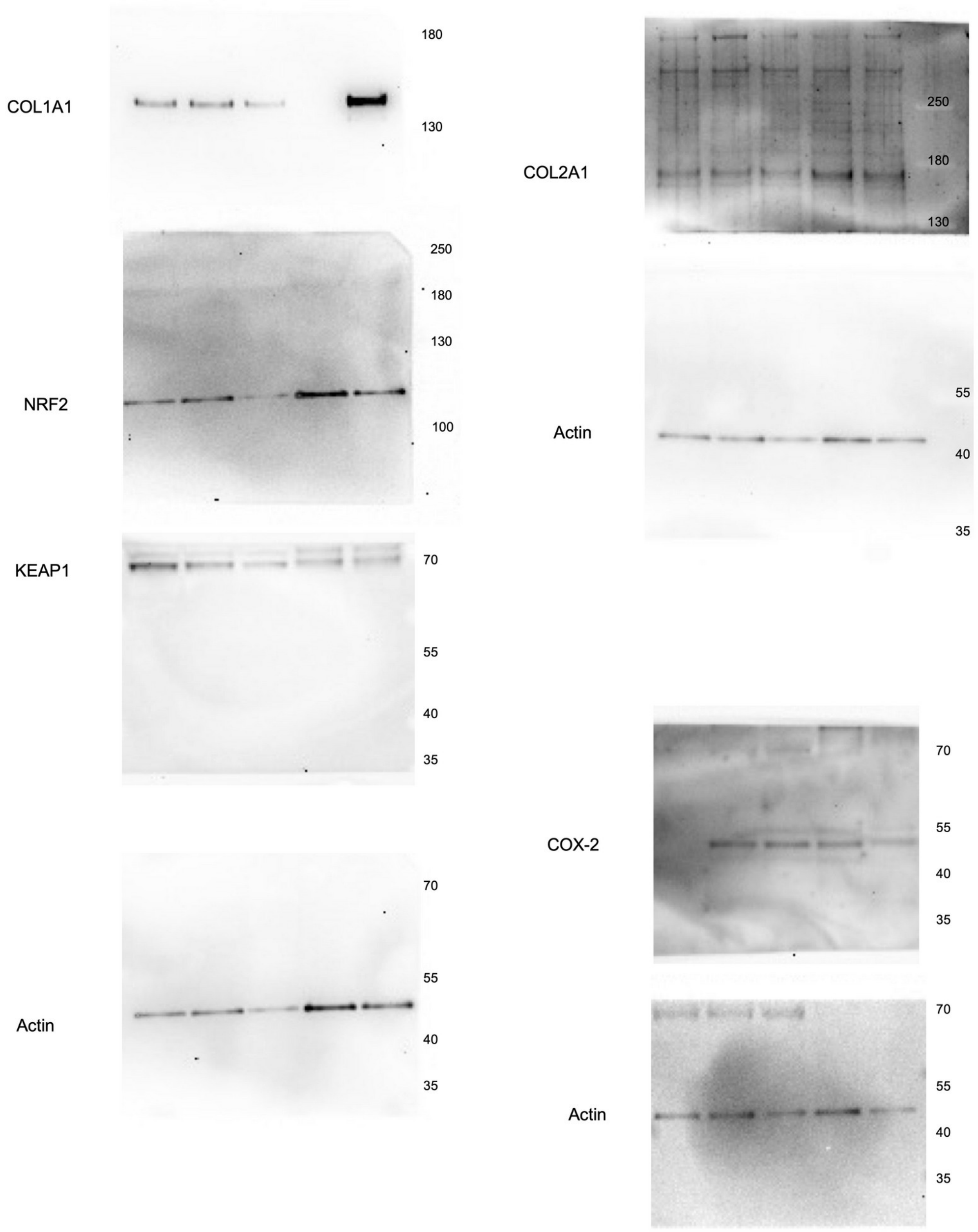
COL1A1
180
130
NRF2
250
180
130
100
KEAP1
70
55
40
35
Actin
70
55
40
35
COL2A1
250
180
130
Actin
55
40
35
COX-2
70
55
40
35
Actin
70
55
40
35
