# Supplementary material for: iMSC-derived extracellular vesicles and their miRNA cargo influence inflammation and oxidative damage in an in vitro osteoarthritis model
Source: Cell Death Dis. 2026 Jun 24;17(1):781. doi: 10.1038/s41419-026-09033-0 (PMC13547349; doi:10.1038/s41419-026-09033-0)
Supplement: Supplementary file 1 [file 41419_2026_9033_MOESM8_ESM.docx]

**Figure S1. Long-term iMSCs SD1, SD2, SD3 iMSC and iEVs characterization. A** Growth kinetics for SD1, SD2, and SD3 along culture time. Data are representative of 3 independent experiments. **B** Quantification of protein content on iEVs using BCA assay. Data normalized to the cell number (μg/106 cells). Data are represented as mean ± (n=3). One-way ANOVA, * p-value < 0,05, ** p-value < 0.01, *** p-value < 0.001, **** p-value < 0.0001. **C** Representative NTA size distribution analysis of iEVs isolated from SD1, SD2, and SD3 at P8, P12, and P16 (n=3).

**Figure S2. Non-conventional Flow Cytoemtry iEVs analysis gating strategy** **A** Representative CFDA-SE staining used to identify intact vescicles. EVs were stained with CFDA-SE at 4°C as control (left) to define the appropriate dimensional gate considering the EVs stained with CFDA-SE at room temperature (RT, right). Red areas identify CFDA-SE positive events. **B** Representative histograms reporting flow cytometry analysis of iEVs isolated at P12 and P16 from SD1 (red), SD2 (green), and SD3 (blue). Histogram curves identify cells reacting with CD9, CD63, and CD81-specific antibodies. The area under the gray curve identifies the reaction of the cells with the corresponding non-reactive immunoglobulin of the same isotype (isotype control). Data are representative of three independent experiments.

**Figure S3. iEVs miRNA cargo attenuates ROS accumulation in inflamed hACs.** Representative histogram reporting flow cytometry analysis of intracellular ROS of OA hACs treated with Scramble miRNA (yellow) and selected miRNAs (orange). The light gray curve represents the negative control and the dark gray curve positive control.

**Figure S4. NRF2 expression is affected by miRNA transfection. A** Quantification of NRF2 protein expression in western blots of OA hACs transfected with 100nM hsa-miR17- 5p, hsa-miR20-5p, hsa-miR21-5p, hsa-miR29a- 3p, hsa-miR29b-3p, hsa-miR29c-3p, or with scramble control. Data are represented as mean ± SD (n=3). One-way ANOVA, * p-value < 0.05, ** p-value < 0.01. **B** Quantification of KEAP1 protein expression in western blots of OA hACs transfected with 100nM hsa-miR17- 5p, hsa-miR20-5p, hsa-miR21-5p, hsa-miR29a- 3p, hsa-miR29b-3p, hsa-miR29c-3p, or with scramble control. Data are represented as mean ± SD (n=3). One-way ANOVA, **** p-value < 0.0001. **C** Quantification of NRF2 protein expression in Western blot of OA hACs transfected with, 100nM miRNAs pool or with scramble control. Data are shown as mean ± SD, (n=3). One-way ANOVA, * p-value < 0.05. CTR: Negative control. **D** Quantification of KEAP1 protein expression in Western blot of OA hACs transfected with, 100nM miRNAs pool or with scramble control. Data are shown as mean ± SD, (n=3). One-way ANOVA. CTR: Negative control.

**Figure S5. miRNAs pool shows synergic anti-fibrotic effects. A** Representative image of COL1A1 staining on OA hACs transfected with SD1 iEVs, 100nM miRNAs pool or with scramble control. Scalebar represent 100 µm. **B** Quantification of COL1A1 positive cells. Data are represented as mean ± SD (n=3), One-way ANOVA. **** p-value < 0.0001. CTR: Negative control.

**Figure S6. miRNAs pool shows synergic pro-chondrogenic effects. A** Representative image of COL2A1 staining on OA hACs transfected with SD1 iEVs, 100nM miRNAs pool or with scramble control. Scalebar represent 100 µm. **B** Quantification of COL2A1 total cell fluorescence. Data are represented as mean ± SD (n=3), One-way ANOVA. ** p-value < 0.01 **** p-value < 0.0001. CTR: Negative control.

**Figure S7. miRNAs pool shows synergic anti-inflammatory effects. A** Representative image of COX-2 staining on OA hACs transfected with SD1 iEVs, 100nM miRNAs pool or with scramble control. Scalebar represent 100 µm. **B** Quantification of COX-2 positive cells. Data are represented as mean ± SD (n=3), One-way ANOVA. **** p-value < 0.0001. CTR: Negative control.
